# On Lithotrity

**Published:** 1830-07-01

**Authors:** 


### XXXVIII.

### LITHOTRITY.

THIS very interesting subject was brought before The Royal Society, in a communication from Mr. Costello. The Meeting on this occasion was the most crowded of any that took place this season. This gentleman, it is known, had been, till very lately, the associate of the Discoverer of Lithotrity, Dr. Civiale, of Paris. The paper commenced by some general reflections on the progress of this method in France, from which it would appear, that it had been already applied successfully in 200 cases, and that the French Institute had, from its very infancy, made every effort to cause it to be generally adopted. Mr. Costello was desirous to obtain for it the sanction of the Royal Society, and accordingly came forward with his *exposé*. This Paper was drawn up in such a manner as to exhibit the gradations of difficulty which the application of Lithotrity may encounter. Its divisions, which we shall notice presently, were well calculated to answer the ends the author had in view; viz. To set bounds to the enthusiasm of those, who imagine that Lithotrity is applicable to every case of calculous affection; to enable practitioners in general to judge for themselves, as to its applicability in any given case; and lastly to convince those who have the misfortune to be afflicted with stone, of the necessity of applying for relief in time, the method being, in the early stage of the disease, attended with certain success, and with little or no pain. To fulfil these views, Mr. Costello adopted a division of his subject, founded on the normal state of the organs in general, and of the urinary organs in particular, and on their greater or less degree of aberration from that state. We thus have four classes of calculous cases; the first, which Mr. Costello denominates simple cases; the second in which there is commencing organic alterations; the third, in which the organic alterations, are profound; the fourth, in which these alterations are extensive, the general health destroyed, and the calculi are large, or numerous. In the first class, four cases are related in which calculus had existed variously from six to fifteen months. The operation in these cases, is as brilliant as it is safe, and expeditious. Each of the patients is delivered of the stone, in one or two sittings, of from three to five minutes duration, and apparently with no more pain, than is usually produced by common sounding. In these cases, the calculus is of recent formation, the urinary organs are in the normal state, and the general health is unimpaired. The patients undergo no confinement during the treatment; a bath is merely ordered after the operation. When the sittings are to be repeated, they walk about in the interval, and are in no wise affected by what has taken place, further than that the fragments of the stone are voided naturally in the urine. In one of these cases, the patient thinks so lightly of the pain occasioned by the operation, that at the time of the definitive exploration of the bladder, and when it is ascertained that it is quite free of any particle of stone, he exclaims, I have suffered a great deal, but now I shall cease to observe a regimen, for if my disease return, I can be rid of it in five minutes. But unfortunately, says Mr. Costello, the application of Lithotrity does not always take place under such favourable circumstances; on the contrary, the alternative hitherto held out for the cure of stone, the knife, by deterring the majority of the sufferers from resorting to it, until the urinary organs became deeply diseased, and the calculi much larger, creates difficulties for the lithotritist, which all his skill and patience cannot sometimes surmount. When there is simply one, or more calculi of recent formation in the bladder, and the urinary organs are sound, the cure is certain and almost painless. The certainty and painlessness of the operation generally speaking, will be always relative to these conditions.

The interest which Sir Astley Cooper took in the propagation of Lithotrity, is mentioned in a very complimentary manner. This line of conduct, on the part of Sir A. Cooper, is in accordance, with the zeal he has always evinced for the advancement of science. One of the patients in the second class, in which Mr. Costello places cases in which there is commencing organic alteration, was operated on by him in Sir A. Cooper's drawing-room, in the presence of several eminent surgeons. This patient obtains his cure in three sittings of three or four minutes each, attended with very little pain, although his case presents three unfavourable circumstances, namely, a narrow urethra, an enlarged prostate, and a columnated and irritable bladder. After the operation, the patient "girds up his loins," and walks down stairs with Sir A. Cooper and the surgeons who had been present. He smiles, and is as merry as if he had been no more concerned than any of the bystanders; on observing which, Sir Astley exclaims, "Really, gentlemen, all this appears to me quite extraordinary; it is unquestionably the most valuable improvement of modern surgery." In another case in this class, complications, equally, if not more serious, are triumphed over with the same facility. The relation of the case of Admiral Poulden proves this. Mr. Costello here makes some reflections on the high operation of lithotomy, which in his opinion, in cases of large calculus bids fair to be more generally resorted to than it has hitherto been.

In the third class, we have various forms of extensive organic mischief, such as catarrh of the bladder, thickening of its substance, fungous growth within its cavity, enlarged prostate, irritability, paralysis of the bladder, incontinence of urine, &c. Even under these untoward circumstances, says Mr. Costello, lithotrity will often succeed. But its application requires the greatest care and caution; and the lithotritist must rather feel his way, than precipitately rush upon the operation under such circumstances. Mr. C's plan, when the irritability is excessive and the health broken, consists in the free exhibition of opium, by the mouth or anus, the application of leeches to the perineum, followed by anodyne fomentations, and the use of the hip or general tepid bath. When the opium disagrees, he employs hyosciamus, conium, &c. To this are added dilution with mucilaginous drinks, a mild regimen and repose on a sofa; alkaline medicines are also employed. When the parts are quieted, he proceeds with the operation in the same manner, as if it were a simple case. In this series we find the case of Mr. Hall, of Dartford, in whom the capacity of the bladder is diminished, the prostate tumefied, the disease is of several years standing, and there is frequent testicular enlargement. The calculus is of the size of a large walnut, and requires seven sittings for its entire destruction and extraction. Mr. Hall was consulted by another gentleman, who subsequently became a patient of Mr. Costello. He quieted his fears by assuring him, there was nothing very terrible in this operation, and that his life ran no risk by it. The gentleman who had been thus re-assured by Mr. Hall, was Mr. Bowdery, a respectable bookseller, in Oxford-street. He was present at the Meeting of the Royal Society, and exhibited, in a tortoise-shell box, the calculus, reduced to powder, of which he had been delivered, he appeared to enjoy excellent health. Another case in this class, is that of Mr. Kearn, parish priest of Rathfarnham, near Dublin, aged 73. In this case the bladder was irritable, the prostate enormously enlarged, the bladder contained several calculi, and catheterism had been several times followed by the eruption of scorbutic blotches on the lower extremities. In the next case, there is thickening of the bladder, incontinence of urine, calculus in the bladder almost from infancy, (the patient being then 19 years old) with extreme marasm. The next is the case of Mr. Bowdery, which exhibits in a striking manner, one of the most formidable complications of stone in the bladder, as well as the skill and tact by which the difficulties it presented were overcome. The bladder contained several calculi, the prostate was enlarged, and there was a fungous growth at the neck of the bladder. Mr. Costello relates this case with great clearness and effect, and closes it by a few very sensible observations on the nature of fungous growths in the bladder in general. He also alludes to the influence which the pressure of his instruments exerts in effecting the resolution of those enlargements of the prostate, which so frequently accompany the advanced stages of calculous disease. It is true, he says, that we cannot determine *à priori* that this resolution will be effected; but it is quite certain that it will take place in a great number of cases, and that it will be favoured, as in other anormal enlargements of tissues, by previous local bleeding. The last case in this series is that of J. V. Batley, a young painter of considerable talent. Mr. Batley had laboured under symptoms of stone for ten years. The calculus had attained the size of a hen's egg, the largest which Mr. Costello's instruments can grasp. There was intense catarrh of the bladder; notwithstanding these unfavourable circumstances, Mr. Costello was perfectly successful. The fourth class embraces cases in which the size, the number of the calculi, the constitutional disturbance, and the organic alteration, render the application of lithotrity inadmissible.

The reading of this paper excited the deepest interest. Mr. Costello's object was to point out the cases favourable for lithotrity, and at the same time the limits within which its application ought to be restrained. His plan was simple and intelligible, and in coming forward thus early, he will have essentially assisted the enquiries of the public and the profession on this very important operation. To us it is quite clear that, after the lapse of some years, when these complicated cases shall be less numerous, and less numerous they must become, because sufferers will now apply for the new remedy, on the earliest appearance of symptoms, lithotomy will be very rarely performed. Mr. Costello is an innovator; but he is so in an open, candid, and scientific manner. On this occasion the thanks of the Royal Society were voted to him for his valuable communication. He subsequently demonstrated the action of his instruments in the library of the Institution.

